# Hypertrophic Lichen Planus Mimicking Verrucous Lupus Erythematosus

**DOI:** 10.7759/cureus.3555

**Published:** 2018-11-06

**Authors:** Ryan R Riahi, Philip R Cohen

**Affiliations:** 1 Dermatology, DermSurgery Associates, Sugar Land, USA; 2 Dermatologist, San Diego Family Dermatology, San Diego, USA

**Keywords:** cutaneous, erythematosus, hypertrophic, lichen, lichenoid, lupus, mimic, mimicking, planus, verrucous

## Abstract

Lichen planus is an inflammatory skin condition that can affect the hair, mucous membranes, nails, and skin. Cutaneous lichen planus typically presents as papules that are planar, polygonal, pruritic, and purple. Subtypes of lichen planus include actinic, annular, atrophic, eruptive, follicular, hypertrophic, inverse, linear, palmoplantar, pemphigoides, pigmentosus, ulcerative, vesiculobullous, and vulvovaginal. The various clinical presentations of lichen planus can mimic other dermatologic conditions. A 63-year-old woman, who presented with pruritic, hyperkeratotic plaques on the lower legs of two years duration, is described; her lesions were morphologically suggestive of verrucous lupus erythematosus. However, an examination also revealed purple papules on the wrists and white, reticulated patches on the bilateral buccal mucosa. Biopsies demonstrated lichenoid dermatitis while laboratory studies for systemic lupus erythematosus were negative. A correlation of the clinical presentation, pathology, and laboratory studies established a diagnosis of hypertrophic lichen planus. The clinical mimickers of hypertrophic lichen planus are reviewed and the therapeutic treatments for this condition discussed.

## Introduction

Lichen planus is a chronic inflammatory and immune-mediated condition of unknown etiology that can affect the hair, mucosa, nails, and skin [1-2]. Classic lichen planus typically involves the flexor extremities and presents as purple, flat-topped papules that are pruritic. Oral lesions can present alone or in conjunction with the cutaneous lesions of lichen planus.

The cutaneous features of lichen planus can present with various morphologies and can mimic other dermatologic conditions [1-5]. In addition, there are several clinical variants of lichen planus: actinic, annular, atrophic, eruptive, follicular, hypertrophic, inverse, linear, palmoplantar, pemphigoides, pigmentosus, ulcerative, vesiculobullous, and vulvovaginal [1-2]. Also, albeit less common, lichen planus may present as part of an overlap syndrome with either lichen sclerosis or subacute cutaneous lupus erythematosus [4-5].

A 63-year-old woman with hypertrophic lichen planus, whose leg lesions mimicked verrucous lupus erythematosus, is described. The clinical mimickers of hypertrophic lichen planus are reviewed. Also, treatment options for this condition are discussed.

## Case presentation

A 63-year-old healthy woman presented for the evaluation of an itchy rash on the lower legs that developed over a period of two years. She had not initiated any new medications. Her medical history was only significant for hypothyroidism for which she took levothyroxine daily.

**Figure 1:**
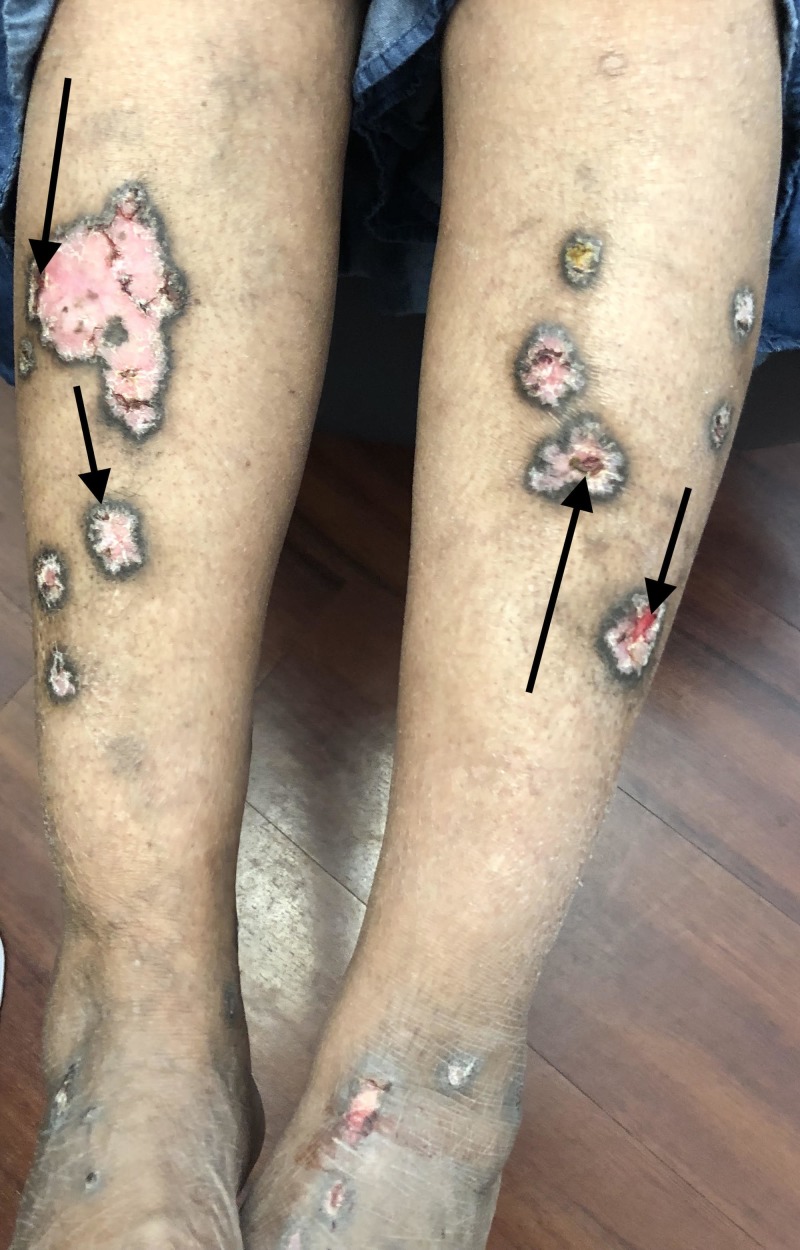
Hypertrophic lichen planus presenting as intact and ulcerated plaques on the legs Multiple, hypertrophic, pink plaques with peripheral hyperpigmentation are symmetrically distributed on the lower extremities of a 63-year-old woman. Some of the hypertrophic lichen planus lesions are ulcerated (arrows).

**Figure 2:**
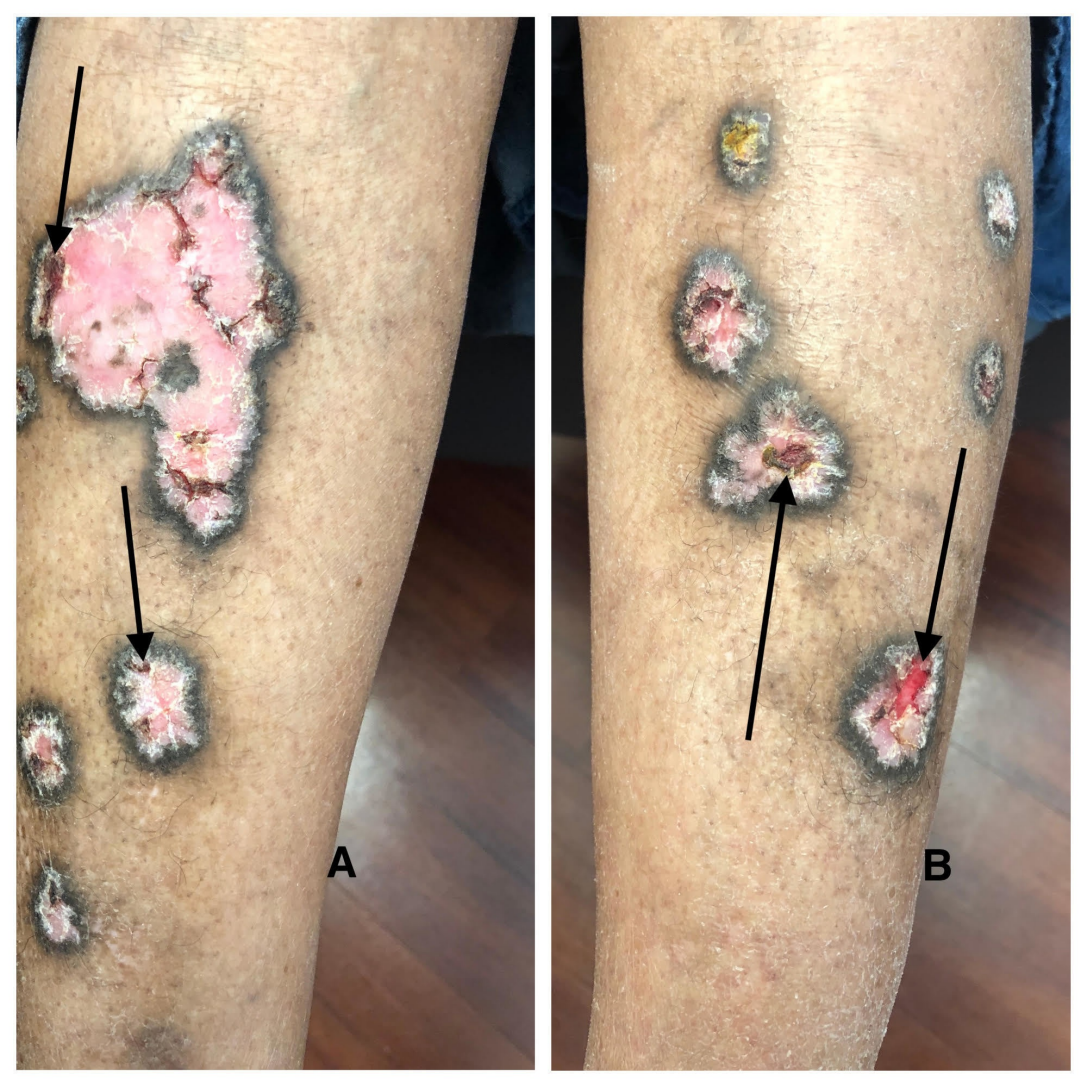
Closer view of the hypertrophic lichen planus on the distal legs Hypertrophic lichen planus appears as hypertrophic pink plaques with peripheral hyperpigmentation on the right (A) and left (B) distal leg. Some of the plaques have peripheral (A) or central (B) ulcerations (arrows) present.

A complete examination of her skin and mucous membranes was performed. The distal legs showed pink plaques with peripheral hyperpigmentation (Figures 1-2).

**Figure 3:**
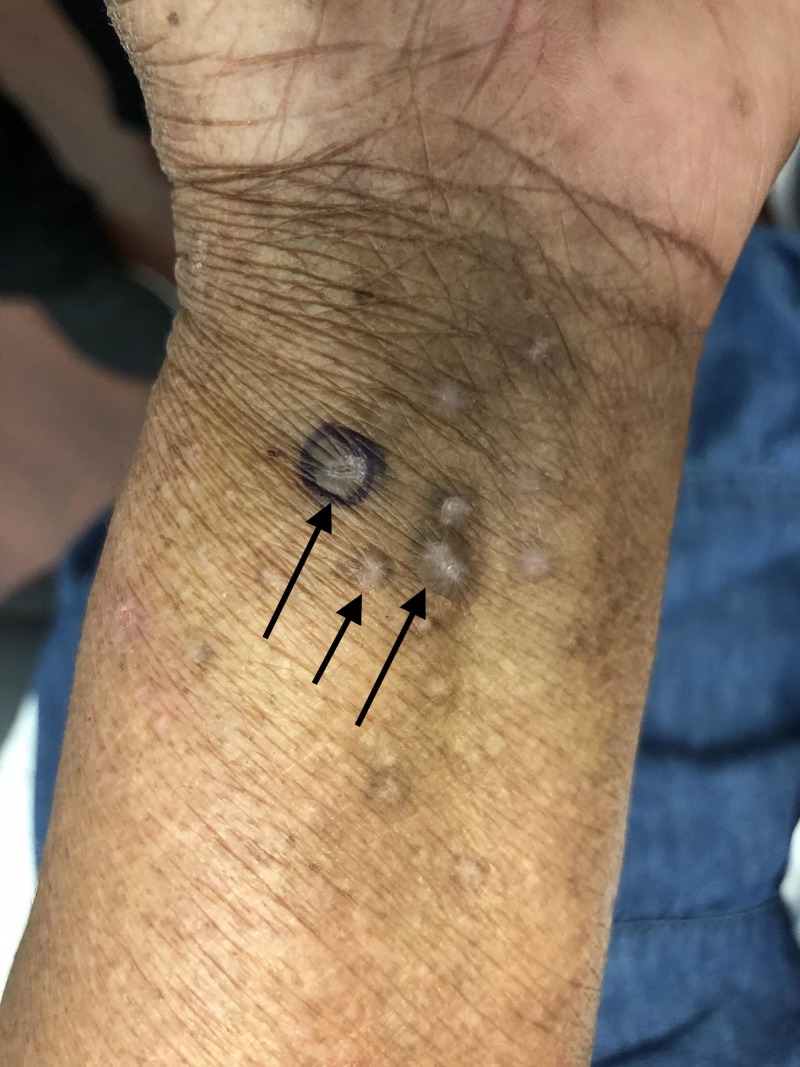
Lichen planus lesions on the left wrist Purple, flat-topped papules (arrows) of lichen planus are present on the left flexor wrist of the 63-year-old woman. The lesion surrounded by purple ink was biopsied for pathologic evaluation.

Purple, flat-topped papules were also present on both wrists (Figure 3). In addition, white, reticulated patches were present on the bilateral buccal mucosa.

Skin biopsies of her left wrist and her right lower leg were performed. They showed hyperkeratosis with an inflammatory infiltrate predominantly composed of lymphocytes present in a lichenoid distribution along the dermal-epidermal junction with apoptotic keratinocytes. These features were considered to be those of lichenoid dermatitis and most consistent with lichen planus.

Antinuclear antibody and double-stranded deoxyribonucleic acid (DNA) antibody tests were performed to evaluate for systemic lupus erythematosus; these serologies were negative. The review of systems was negative for oral ulcerations, joint pain or swelling, and alopecia. Correlation of the clinical findings, pathology, and laboratory studies established a diagnosis of hypertrophic lichen planus.

The patient was treated with topical clobetasol 0.05% cream applied daily to the lesions on her legs as well as oral prednisone 40 milligrams daily for two weeks. At the two-week follow-up, her condition had improved; therefore, over the next month, the daily systemic prednisone was slowly tapered and she continued to apply the topical corticosteroid cream. At her subsequent follow-up appointments, a continued improvement of her condition was observed.

## Discussion

Hypertrophic lichen planus typically presents with hyperkeratotic papules, plaques, and nodules on the lower extremities [1]. Lesions of hypertrophic lichen planus can also involve the upper extremities and trunk, and present in a generalized manner. The condition tends to be pruritic, symmetrical, and chronic. It is postulated that the Koebner phenomenon (whereby lesions occur at sites of skin trauma) contributes to the development of new lichen planus skin lesions given the intense pruritus and tendency for chronic rubbing and scratching by affected individuals [2].

Hypertrophic lichen planus can mimic other dermatologic conditions (Table 1) [1-2,6-7].

**Table 1:** Clinical mimickers of hypertrophic lichen planus

Diagnosis	References
Amyloidosis	[1]
Kaposi sarcoma	[1]
Lichen simplex chronicus	[1]
Prurigo nodularis	[1,7]
Psoriasis vulgaris	[1]
Squamous cell carcinoma	[2,6]
Stasis dermatitis	[1]
Verrucous lupus erythematosus	Current report

Indeed, other pruritic conditions, such as prurigo nodularis and lichen simplex chronicus, may present with similar appearing lesions. Squamous cell carcinoma can also mimic hypertrophic lichen planus; in addition, squamous cell carcinoma can develop in hypertrophic lichen planus lesions [2,6]. The clinical presentation of our patient’s leg lesions mimicked those of verrucous lupus erythematosus.

A complete examination of the skin and mucous membranes—including the nails, oral cavity, and scalp—should be performed when the diagnosis of hypertrophic lichen planus is entertained. In addition, the skin lesions should be biopsied to confirm the diagnosis. Laboratory testing for lupus serologies should be considered if lupus erythematosus or a lichen planus-lupus erythematosus overlap syndrome is suspected.

Treatment options for hypertrophic lichen planus are similar to those of classic lichen planus [1-17]. First-line therapies include either topical corticosteroids (alone or under occlusion), intralesional triamcinolone, and/or oral prednisone. Alternative treatments are listed in Table 2 [1-15].

**Table 2:** Treatments for hypertrophic lichen planus

Treatment	References
Acitretin	[8,14]
Azathioprine	[12]
Biologics (adalimumab, alefacept, efalizumab)	[10,13,15]
Corticosteroids (intralesional, oral and topical)	[1-14]
Cyclosporin	[10-11]
Dapsone	[13]
Enoxaparin	[14]
Griseofulvin	[14]
Hydroxychloroquine	[9,13]
Lower molecular weight heparin	[10,13]
Methotrexate	[9]
Metronidazole	[10,13]
Mycophenolate mofetil	[9]
Phototherapy	[8,10,14]
Sulfasalazine	[14]

Our patient’s hypertrophic lichen planus improved after she received concurrent topical clobetasol 0.05% cream and oral prednisone, starting at a dose of 40 mg taken daily with a taper over the course of six weeks.

## Conclusions

Lichen planus and its subtypes can have various clinical morphologies and presentations. Hypertrophic lichen planus can mimic other dermatologic conditions as well as, albeit rarely, present as part of an overlap syndrome with either lichen sclerosus or lupus erythematosus. Not only a complete examination of the skin and mucous membranes but also a skin biopsy—in addition to possible laboratory testing—may be necessary to confirm the diagnosis of hypertrophic lichen planus. Treatment with topical, intralesional, or oral corticosteroids is the first line of therapy for this condition. Additional treatment measures may be considered for those patients with hypertrophic lichen planus who do not respond to corticosteroid therapy.
